# Geographic distributions shape the functional traits in a large mammalian family

**DOI:** 10.1002/ece3.8039

**Published:** 2021-08-18

**Authors:** Yingjie Ma, Meng Wang, Fuwen Wei, Yonggang Nie

**Affiliations:** ^1^ Key Laboratory of Animal Ecology and Conservation Biology Institute of Zoology Chinese Academy of Sciences Beijing China; ^2^ University of Chinese Academy of Sciences Beijing China; ^3^ Center for Excellence in Animal Evolution and Genetics Chinese Academy of Sciences Kunming China

**Keywords:** ecological adaptation, functional traits, geographic distribution, phylogeny, reproduction, Ursidae

## Abstract

Traits of organisms are shaped by their living environments and also determined in part by their phylogenetic relationships. For example, phylogenetic relationships often affect the geographic distributions of animals and cause variation in their living environments, which usually play key roles in the life history and determine the functional traits of species. As an ancient family of mammals, bears widely distribute and have evolved some specific strategies for survival and reproduction during their long‐term evolutionary histories. Many studies on the ecology of bears have been conducted in recent decades, but few have focused on the relationships between their geographic distributions and ecological adaptations. Here, using bears as a model system, we collected and reanalyzed data from the available literatures to explore how geographic distributions and phylogenetic relationships shape the functional traits of animals. We found a positive relationship between phylogenetic relatedness and geographic distributions, with bears distributed in adjacent areas applying more similar strategies to survive and reproduce: (a) Bears living at high latitudes consumed a higher proportion of vertebrates, which may provide more fat for adaptation to low temperatures, and (b) their reproduction rhythms follow fluctuations in seasonal forage availability and quality, in which bears reach mating status from March to May and give birth in approximately November or later.

## Graphical abstract

We used bears as a model system, and collected and reanalyzed data from the available literature to explore how geographical distribution shapes the functional traits of animals. This is the first time to use the species within a family to explore the relationships between geographic distribution and functional traits of animals and demonstrate the ecological adaptations of animals with close phylogenetic relationships.

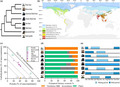

## 1 INTRODUCTION

Animals often face variability in environmental conditions including but not limited to latitude, longitude, temperature, humidity, and the plant growth cycle (Southwood, 1977). Species have evolved adaptations to heterogeneous living environments in order to meet their specific needs, maintain homeostasis, and ensure that they have a sufficient body condition to survive and reproduce (Conaway, 1971; Lane et al., 2012; Raubenheimer et al., 2012; Rusak & Zucker, 1975). With the rapid rise of phylogenetic knowledge, there is a growing appreciation of the extent and possible roles of phylogeny (Donoghue, 2008; Vamosi et al., 2009). Together with environmental explanations, we may able to have a better understanding of ecological adaptations of animals. Thus, analyses of the evolution of life‐history strategies depend upon an understanding of both phylogenetic constraints and the ecological variables that influence their expression (Cavender‐Bares et al., 2009; Thom et al., 2004).

Life histories of animals are shaped by reproduction strategies, a decision with major fitness consequences, which is crucial in many species because the reproductive success of individuals, as well as annual population growth, relies on it (Healy et al., 2019; Price et al., 1988). Most of the animals do not reproduce year‐round but instead display distinct seasonal peaks in reproductive activity (Brown & Shine, 2006) because a mismatch between birth timing and optimal forage availability will decrease animals’ annual recruitment (Post & Forchhammer, 2008). Forage availability limits the timing of reproduction in birds (Dunn et al., 2011), reptiles (van Marken Lichtenbelt & Albers, 1993), mammals (Plard et al., 2014), fish (Caceres et al., 1994), and invertebrates (Kennish, 1997), especially by limiting a female's ability to conceive (Bomford, 1987; Cook et al., 2001). For example, an early breeding time and a large clutch size of blue tits (*Parus caurleus*) were associated with an early and abundant food supply (Blondel et al., 1993). Verreaux's sifaka (*Propithecus verreauxi verreauxi)* mated when food availability was high, and also wean an infant when they had higher body mass during the mating season (Lewis & Kappeler, 2005).

Nutrition, as an indispensable factor of forage besides forage availability, is a core factor that affects the behavior and physiology of animals (Costantini, 2014), and it is one of the most important links between geographic range patterns and animal ecology (Brown, 1995; Stevens, 1989). It is well known that nutrition is highly variable and commonly limited during winter (Parker et al., 2009); therefore, seasonal limitation of nutrition is a challenge that animals face. This nutritional restriction has influenced a variety of life processes in animals, including reproduction (Williams et al., 2017), survival, and population dynamics (Geiser, 2013; González‐Bernardo et al., 2020; Wallmo et al., 1977). To survive and reproduce, animals thus need to adapt yearly environmental variations (McNamara et al., 2011). For instance, bears will store as many lipids as they can before winter and enter hibernation to survive under nutritional restriction (González‐Bernardo et al., 2020). Black‐tailed deer need to store abundant digestible energy during summer, which is very critical for winter survival as well (Parker et al., 1999). The seasonal nutrition strategy is one of the manifestations of adaptive evolution in life‐history strategies.

As the major macronutrients in the diet, proteins, carbohydrates, and lipids are three energy resources that sustain an organism's various functions (Institute of Medicine, 2005). As previously reported, the protein‐leverage hypothesis predicts relatively constant protein‐dominated macronutrient balancing (Felton et al., 2009; Simpson & Raubenheimer, 2005; Srensen et al., 2012). Reproduction may be affected by protein (Barboza & Parker, 2009) and maximized at a higher protein–carbohydrate intake ratio (Lee et al., 2008; Ma et al., 2019). However, life span is maximized at a lower protein–carbohydrate intake ratio (Lee et al., 2008; Solon‐Biet et al., 2014, 2015). Although it is assumed that terrestrial food webs are built on the basis of nitrogen limitation, nonprotein energy is a factor that should not be ignored (Rothman et al., 2011). Lipid accumulation is essential for animals to survive over a prolonged period when food resources are not available (Humphries et al., 2002, 2003; Young, 1976) and plays a significant role in fetal and newborn growth (Hackländer et al., 2002; Herrera, 2002).

The bear family is widely distributed worldwide (Penteriani & Melletti, 2020), with the polar bear (*Ursus maritimus*) exhibiting the northernmost distribution (Amstrup, 2003) and the spectacled bear (*Tremarctos ornatus*) exhibiting the southernmost distribution (García‐Rangel, 2012). Under different environmental conditions, there is marked character segregation between species within the family (Christiansen, 2008; Sacco & Van Valkenburgh, 2004; Spady et al., 2007). Although bears belong to Carnivora, they have adopted diverse foraging habitats during their long‐term evolutionary histories. Most bears are omnivores that live primarily on plant material and secondarily on some insects, fish, and mammals (Penteriani & Melletti, 2020). At the extremes, the polar bear and the giant panda (*Ailuropoda melanoleuca*) are purely carnivorous and herbivorous, respectively (Best, 1985; George et al., 1985; Nie et al., 2019; Stirling & McEwan, 1975). Owing to seasonal nutrition availability, brown bears (*Ursus arctos*), American black bears (*Ursus americanus*), and Asiatic black bears (*Ursus thibetanus*) enter hibernation during winter, and polar bears go through a phase of fasting in summer (Nelson et al., 1983; Robbins et al., 2012). Except for sun bears (*Helarctos malayanus*), which breed year around (Frederick et al., 2012), bears mainly breed in approximately spring to summer and give birth from fall to winter (Eiler et al., 1989; García‐Rangel, 2012; Garshelis et al., 1999; Ramsay & Stirling, 1986; Spady et al., 2007; Steyaert et al., 2012; Wei & Hu, 1994). Therefore, bears’ morphological diversity, widespread distribution, and variation in life‐history patterns make them ideal for studying the adaptability of species to the conditions in their geographic distributions. With the purpose to determine how phylogenetic and environmental features shape the ecological adaptations of bears, we predict that species with close geographic distribution would have similar functional traits to adapt to similar environments.

## 2 MATERIALS AND METHODS

### 2.1 Macronutrient composition of diets

We collected data from the global range of eight species belong to the family Ursidae from the literatures. To standardize the database, certain criteria were applied for the selection of studies. We required estimates of the dietary proportion of mass that bears consumed to calculate macronutrient compositions. Therefore, studies where foods were originally reported as the proportion of dry mass of diet or were estimable after applying fecal correction factors (CFs) to percent fecal volume or dry weight estimates were included (Baldwin & Bender, 2009; Bojarska & Selva, 2012; Furusaka et al., 2019; Hewitt & Robbins, 1996). We excluded studies where diet composition was assessed in ways other than percent fecal volume and dry mass. Seven food categories were considered: green vegetation, soft mast (fleshy fruits), hard mast (nuts, acorns, and seeds), invertebrates (insects), small mammals (rodents), large mammals (ungulates), and fish (only in brown bears).

The use of CFs is considered to be a suitable and reliable method for bear diet assessment (Coogan et al., 2018). We used the data where possible to obtain the dry matter, and the percent fecal volume was corrected by CFs. For bears with only percent fecal volume data, we applied our chosen CFs to re‐estimate the dry matter proportion in their diets. Averaged different monthly estimates were used as seasonal estimates, and averaged monthly estimates were also used in calculating the annual diet of each bear. There are two sets of groups to classify the seasonal diet estimates based on differences in local climate among bears: (a) spring, summer, fall, and winter; and (b) dry, monsoon, and winter. We used the CFs listed in Hewitt and Robbins (1996) and other literature (Table S1). In short, CFs are applied by multiplying percent fecal volume estimates of food items by their respective CF values (i.e., %Vol * CF). The resulting values were then summed together, and estimates of each food item are presented as a percentage of this sum (Hewitt & Robbins, 1996). CFs are given in Table S1. However, diet composition data were unavailable for some bears.

After obtaining estimates of the percentage of dry matter in diets, we estimated the macronutrient composition (protein, nonstructural carbohydrates, and fat) of each food group using data collected from previous studies and the USDA National Nutrient Database (Table S1). Graminoids and forbs were included in the same green vegetation category. We used estimates of whole carcasses for animal prey, which is more credible than other approaches and does not produce overestimates (Coogan et al., 2014). Then, we averaged macronutrient estimates in each group to obtain the average protein value of soft mast. We were unable to obtain access to the nutritional composition of all reported foods in the literature, and this could induce error in the macronutrient estimates of certain bears. However, it is unlikely to have a serious impact on estimates for species (Remonti et al., 2016; Senior et al., 2016).

### 2.2 Data collection

The geographic distributions of extant bears were obtained from IUCN data (http://www.iucnredlist.org). Using the WorldClim database (http://worldclim.com), the monthly and annual temperatures (°C) and precipitation (mm) from 1950 to 2000 were calculated for each species, and the resolution was 340 km^2^. The nonrandom impact of human activities has had a major impact on the ecology of bears over the past thousand and particularly past hundred years, resulting in the selective loss of ecotypes such as brown bears from the American Southwest (Martin et al., 2010). To take into account the effects of these disturbances, we extracted the human footprint index (Human Footprint 2009, http://wcshumanfootprint.org/, Venter et al., 2016), as a proxy of potential human disturbances. This index measures the cumulative impact of direct and indirect pressures on natural ecosystems from human activities.

### 2.3 Statistical analysis

We used right‐angled mixture triangles (RMTs) to explore the annual macronutrient composition in eight bears, which represents the 3‐component composition of mixtures in a multidimensional nutrient space (Raubenheimer, 2011). Diet composition of six omnivorous bears and seasonal nonprotein energy differences between the panda and other bears were assessed by one‐sample *t* test. Protein energy and nonprotein energy were compared between hibernating and nonhibernating bears with independent *t* tests. These tests were performed using SPSS v.22.0 (IBM Corp.). RMT model, diet composition, and seasonal macronutrient composition were carried out and created with GraphPad Prism software version 5.0 (GraphPad Software Inc.). Cluster analysis by environmental variables, including mean temperature, latitude, mean precipitation, ratio of forest area, and human footprint, was used to assess the similarity of different bear environments. Variables were standardized first to remove the influence of dimension and magnitude (Lu et al., 2019). The distance between variables was measured by Euclidean distance. Then, differences between clusters were tested by the Wilcoxon test. These analysis, tests, and graphs were performed by software R (R version 4.1.0 downloaded from https://www.R‐project.org). To characterize further the relationship between functional traits, phylogeny, and environments, we calculated the Spearman nonparametric rank correlation coefficients for all bears. The correlation in matrix analysis was carried out by using the corrplot package to perform the Pearson correlation analysis through software R (with the corrplot package downloaded from https://cran.r‐project.org/web/packages/corrplot). The correlation coefficient and significance (p‐value) were extracted through programming statements cor and res. Statistical significance was established a priori as *p* < .05.

## 3 RESULTS

### 3.1 Ecological adaptation of bears

**FIGURE 1:**
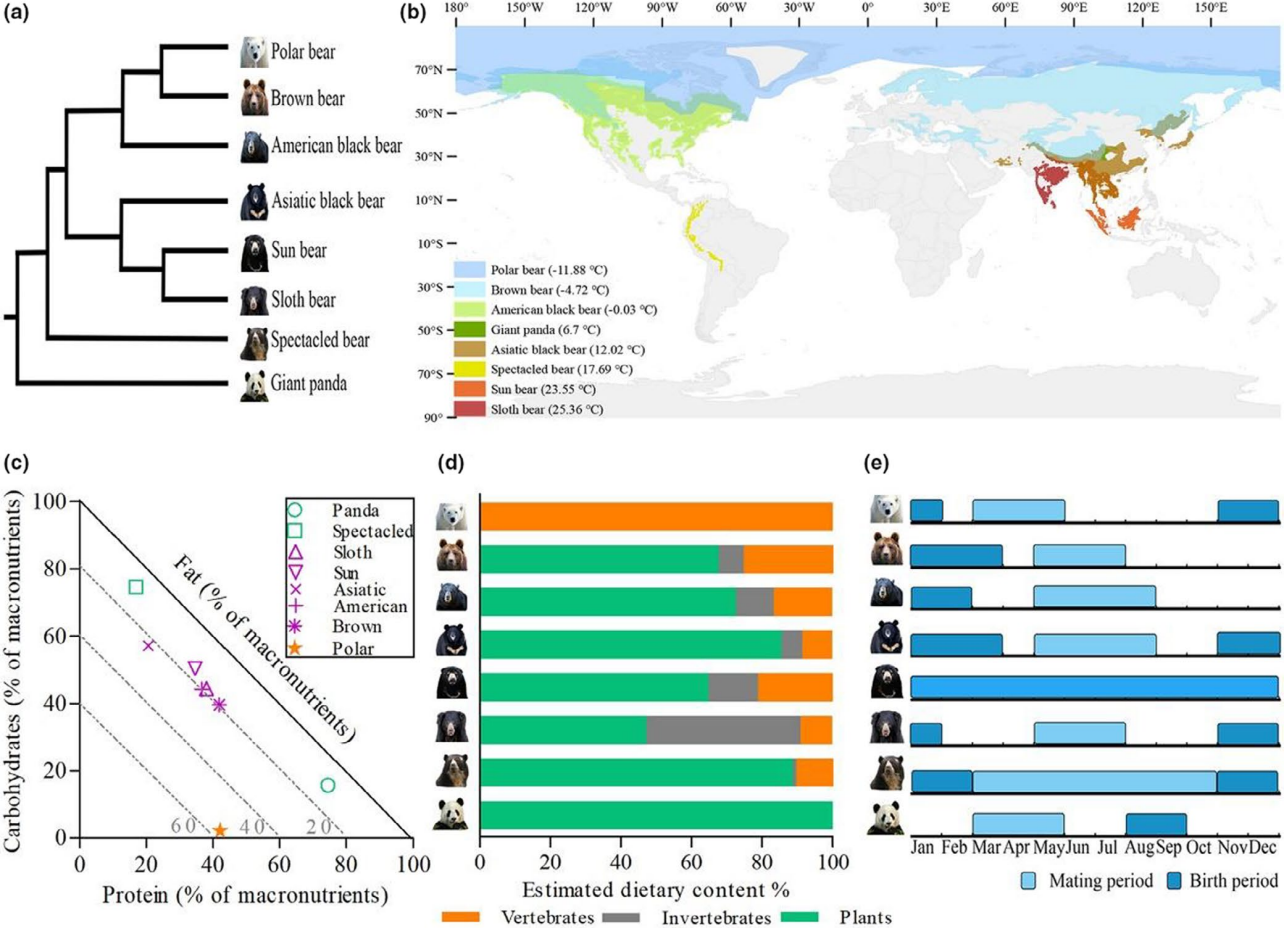
Phylogenetic relationships, geographic distribution, annual macronutrients, estimated dietary contents, and reproduction timing of extant bears. (a) Phylogenetic relationships among the bears constructed using low‐coverage genomes modified from Kumar et al. (2017) and Lammers et al. (2017). (b) Geographic distribution of extant bears according to IUCN data (http://www.iucnredlist.org) and annual mean temperature data with a spatial resolution of 340 km^2^ from WorldClim (http://worldclim.com). (c) Right‐angled mixture triangle showing the macronutrient balance of the estimated diets of bears. Macronutrients are expressed as the percentage of total macronutrients (protein + fat + carbohydrates). The value of fat is inversely related to distance from the origin. (d) Estimated dietary plant, invertebrate, and vertebrate contents of bears. (e) Range of mating and birth period of bears. The sun bear is a polyestrous and nonseasonal breeder

The low‐coverage genome‐based phylogeny provides a clear image of bears' phylogenetic relationships (Figure 1a). The giant panda that belongs to the subfamily *Ailurinae* diverged first, followed by the spectacled bear, a member of the *Tremarctinae*. Within the subfamily *Ursinae*, there are two main monophyletic clades: one consisting of the Asiatic black bear, sun bear, and sloth bear (*Melursus ursinus*), and another consisting of the American black bear, brown bear, and polar bear (Lammers et al., 2017).

Bears are distributed at different latitudes from the Northern to Southern Hemispheres with increased annual mean temperature (Figure 1b; *R* ^2^ = 0.79, *p* = .003). Across geographic gradients, the polar bear, brown bear, and American black bear are widely scattered around higher latitudes, while the Asiatic black bear, sun bear, and sloth bear have smaller ranges with adjacent or partly overlapping distributions. The spectacled bear is the only bear species in South America, and the giant panda has the smallest distribution area, located in the mountains of central China.

The feeding habits of bears vary from herbivory and omnivory to carnivory because of significant environmental variations (Figure 1d). Polar bears live in the Arctic region and consume almost 100% of vertebrates, while giant pandas exclusively feed on bamboos and other bears consume diets with different proportions of plants, invertebrates, and vertebrates depending upon seasonal forage availability. Particularly, among the six omnivorous bears, the sloth bear consumed the lowest proportion of plants (*t *= 6.00, *p* = .004) and the highest proportion of invertebrates (*t* = −16.18, *p* = .000) and the spectacled bear consumed the highest proportion of plants (*t* = −3.43, *p* = .027). The annual macronutrients of bears varied in the RMT model (Figure 1c). The giant panda ingested the highest percentage of protein but low percentages of fat and carbohydrates. The spectacled and polar bears ingested the highest percentages of carbohydrates and fat, respectively. Other bears had similar percentages of fat in their diets but different percentages of protein and carbohydrates.

Given their different distribution areas, climate features, and seasonal forage availabilities, bears exhibited diversified reproductive strategies (Figure 1e). Sun bears are the only polyestrous and nonseasonal breeders distributed in the tropics. Other bears exhibit seasonal reproduction rhythms. That is, the giant panda, spectacled bear, and polar bear approximately reach mating status in March, while other bears reach mating status in May. In contrast to the giant panda, which gives birth in approximately August to September, the other bears mainly give birth in approximately November or later.

### 3.2 Comparison between functional traits and seasonal energy deposition strategies

**FIGURE 2:**
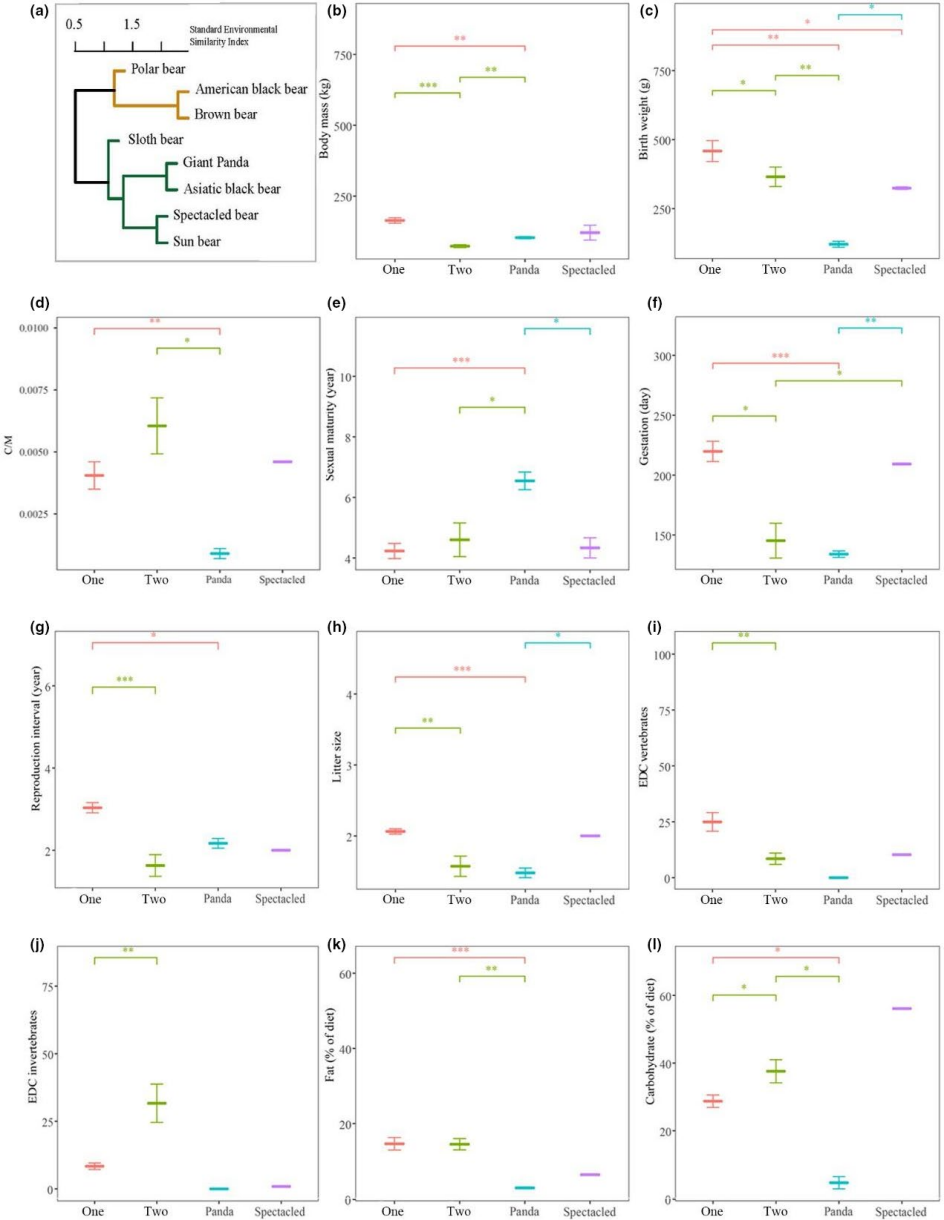
Cluster analyses and comparison of functional traits. (a) Cluster analyses of bears by their environmental variables and comparison between (b) body mass, (c) birthweight, (d) C/M, (e) sexual maturity, (f) gestation, (g) reproduction interval, (h) litter size, (i) EDC vertebrates, (j) EDC invertebrates, (k) fat (% of diet), and (l) carbohydrate (% of diet) between different clusters. Cluster 1 included polar bear, brown bear, and American black bear. Cluster 2 included Asiatic black bear, sun bear, and sloth bear. The giant panda and spectacled bear were displayed separately owing to their special distribution characteristics. Asterisks indicate significant differences (***p < .001; **p < .01; and *p < .05)

Cluster analysis of bears by their environmental variables statistically organized eight bears into a dendrogram (Figure 2a). There are two main clusters: one consisting of the polar bear, American black bear, and brown bear, and another consisting of the sloth bear, giant panda, Asiatic black bear, spectacled bear, and sun bear. There were significant differences between cluster 1 (polar bear, American black bear, and brown bear) and cluster 2 (sloth bear, Asiatic black bear, and sun bear) across their functional traits. The giant panda (only distributes in the mountains of China) and spectacled bear (only distributes in South America) were displayed separately owing to their special distribution characteristics. The body size, birthweight, gestation, reproduction interval, litter size, and EDC (estimated dietary content) vertebrates were higher in cluster 1 than those in cluster 2 (Figure 2b,c,f,g,h,i). However, EDC invertebrates and carbohydrate of diet were both higher in cluster 2 than those in cluster 1 (Figure 2j,l). The giant panda had the smallest birthweight (Figure 2c) and the longest sexual maturity (Figure 2e).

**FIGURE 3:**
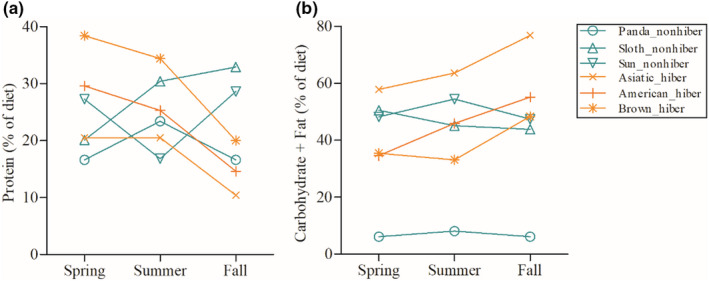
Seasonal energy deposition strategies. (a) Protein energy and (b) nonprotein energy (fat + carbohydrates) of the diet between hibernating and nonhibernating bears. Spring, summer, and fall are equal to the dry, monsoon, and winter periods of the sun bear and Andean bear, respectively

The hibernating bears (Asiatic black bear, American black bear, and brown bear) and the nonhibernating bears (sun bear, sloth bear, and giant panda) exhibited different strategies of energy deposition before winter. From spring to fall, the deposition of nonprotein energy (carbohydrates + fat) of hibernating bears was significantly higher than that of nonhibernating bears (Figure 3b; *t* = 6.32, *p* = .003), while the deposition of protein of the hibernating bears was significantly lower than that of the nonhibernating bears (Figure 3a; *t* = −4.04, *p* = .016). Among all bears, the giant panda ingested the smallest amount of nonprotein energy (Figure 3b) in spring (*t* = 8.72, *p* = .001), summer (*t* = 7.92, *p* = .001), and fall (*t* = 8.14, *p* = .001).

### 3.3 Correlations between functional traits, phylogeny, and environments

**FIGURE 4:**
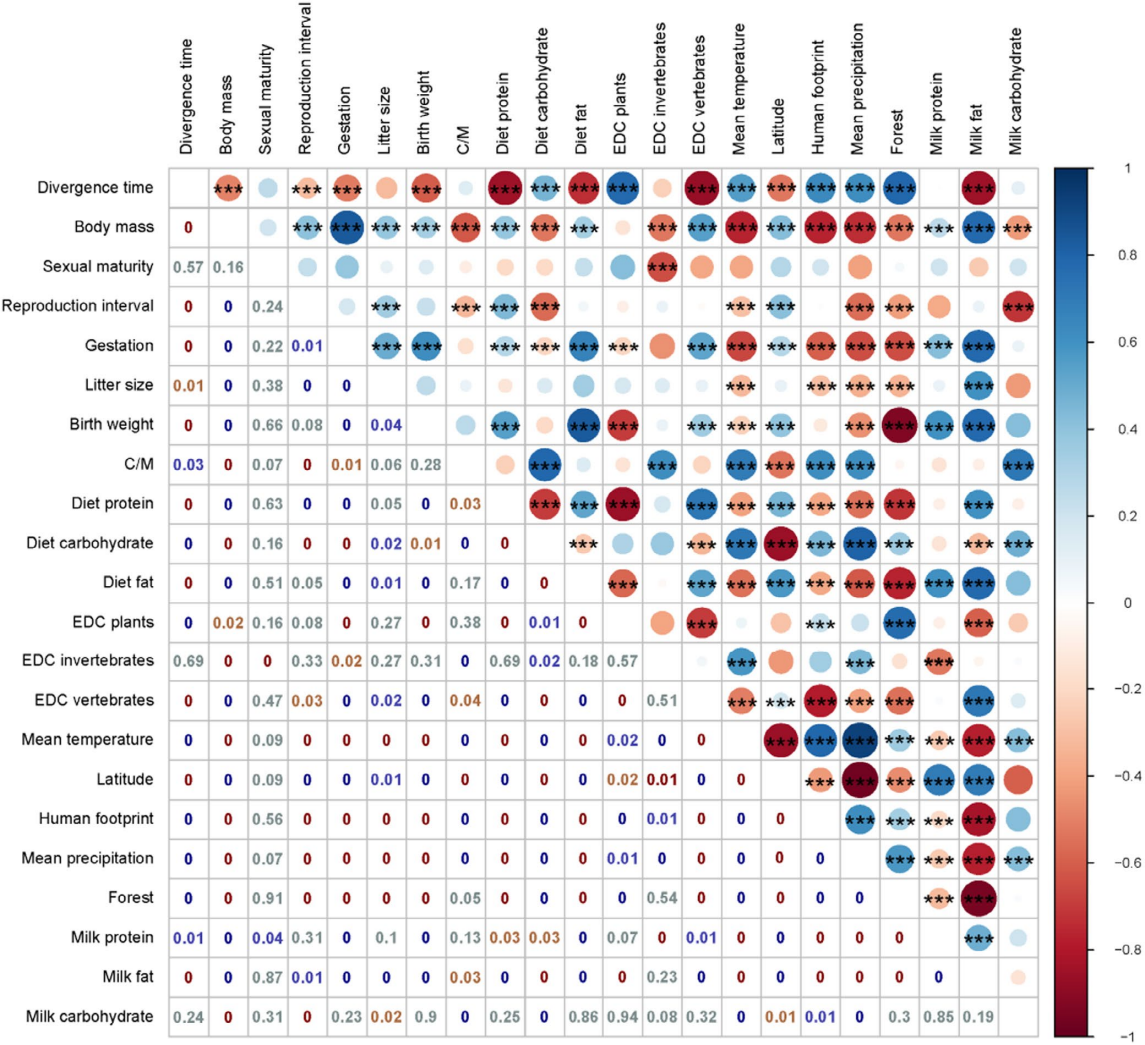
Matrix analysis of the correlation between functional traits and phylogeny and environment. The scale colors denote whether the correlation is positive (closer to 1, dark blue) or negative (closer to −1, dark red). The Spearman nonparametric rank correlation *p*‐values were shown as numbers. Significant correlations were shown (*p*‐value < .001) by 3 asterisks on the circle. C/M, cub birthweight/maternal body mass; EDC, estimated dietary content; forest, ratio of forest area

The correlation strength along with their associated significance levels was indicated in different colors of circles and numbers (Figure 4). The divergence time of bears was positively correlated with environmental variables, only excluding the latitude. High latitude corresponded to low temperature, low precipitation, low human footprint, and low forest area. Bears that distributed in high latitude had the food composition with high EDC vertebrates, low EDC invertebrates, and low EDC plants. Thus, bears distributed in high latitude had high diet protein and fat, but low carbohydrate. After all, these factors were responsible for the large body mass, long gestation, large birthweight, and high milk fat of bears that distributed in high latitude.

## 4 DISCUSSION

### 4.1 Ecological adaptation of bears

Our findings indicate that the phylogenetic relationships are in line with the pattern of different distributions of bears. In the face of various climates, latitudes, and foraging availabilities, the functional traits of bears are different from each other. Our analysis suggests that the vertebrate proportion of a bear's diet is positively related to the latitude and negatively related to the temperature. Specifically, the polar bear, brown bear, and American black bear are mainly found at high latitudes. To cope with the low temperature, the polar bear mainly consumes aquatic animals with high percentage of fat such as ringed seals (*Phoca hispida*), bearded seals (*Erignathus barbatus*), harp seals (*Pagophilus groenlandica*), and beluga whales (*Delphinapterus leucas*) (Stirling & Archibald, 1977; Thiemann et al., 2008). For the brown bear and American black bear, the long cold winter is a period of food shortage that must be endured. These bears require more high‐energy food than other bears for survival (González‐Bernardo et al., 2020). Hence, there is a high proportion of vertebrates in their diets. On the other hand, the Asiatic black bear, sun bear, and sloth bear are distributed in middle‐ or low‐latitude areas with abundant seasonal forage availability. Thus, they have a greater food selection and diversified food compositions with a lower proportion of vertebrates. Likewise, the spectacled bear and giant panda feed on a large or full proportion of plants.

Bears with different forage availabilities have different nutritional strategies, including fat maximization (Stirling & McEwan, 1975), carbohydrate maximization (Figueroa, 2013), and protein maximization (Nie et al., 2019). Our analysis also demonstrates that the vertebrate proportion is positively related to diet protein and fat. In the balance among annual protein, carbohydrates, and fat, the polar bear has the highest fat intake among the eight bears because the aquatic animals they feed on are high in fat (Stirling & McEwan, 1975). The other species have almost the same proportions of fat ingestion, but their protein intake increases with the proportion of vertebrates in the diet. Because of the high percentage of protein energy from bamboos (Nie et al., 2019), the giant panda has the highest protein intake. On the contrary, because of the high percentage of carbohydrate energy from the soft mast, the spectacled bear has the highest carbohydrate intake (Figueroa, 2013).

### 4.2 Comparison of functional traits

Bears distributed in adjacent areas show similar patterns of nutrition, survival, and reproduction strategies. The polar bear, brown bear, and American black bear locate in high latitude with low temperature and precipitation. They have a higher proportion of vertebrates in diet, which means they have a higher intake of protein and fat. Therefore, they have larger body mass, gestation, birthweight, and litter size than other bears. High energy intake of protein and fat is critical to cope with low temperature (González‐Bernardo et al., 2020; Parker et al., 1999). Longer gestation and larger litter size are considered to have a higher survival rate for their offspring (Ronget et al., 2018). Studies have also demonstrated that higher birthweights can effectively improve survival rate, especially in mammals (Karimi et al., 2018; Ronget et al., 2018). Increasing body mass may increase energy conservation and probability of survival (Schorr et al., 2009). Fat is considered the main component of body reserves (Bennett et al., 2015; Monteith et al., 2014), and it allows large individuals to survive over periods of food shortage.

The comparison between and analysis of hibernating and nonhibernating bears show that there are apparent seasonal differences in nutrient accumulation. Hibernating bears acquire more protein than nonhibernating bears in spring to make preparations for breeding. In fall, hibernating bears will accumulate vast amounts of nonprotein energy, which is more suitable than protein energy for storage in winter. Additionally, hibernating bears give birth during hibernation. Based on our results, dietary fat is essential for both of the female and cubs’ survival. Bears have delayed implantation, and their virtual gestation period is 6–8 weeks. Then, the females give birth in dens and nurse cubs with their own accumulated energy in winter (Farley & Robbins, 1995). By nursing small neonates instead of maintaining developing fetuses, a fasting female could use her fat stores and avoid the loss of body protein resulting from gluconeogenesis (Ramsay & Dunbrack, 1986). Neonates are able to catabolize fatty acids within a few hours of birth through milk (Hahn, 1979), and fat is a crucial element for neonatal brain and nerve development (Crawford, 1993).

Ursids inhabit a wide range of habitats on four continents, including the arctic, temperate, and tropical zones. The wide distributions explain their survival and reproductive strategies that are in harmony with seasonal environmental conditions. This is the first time to reveal a correlation between the geographic distributions and functional traits of animals belonging to the same family, which provides insights into understanding of the ecological adaptations of animals with close phylogenetic relationships living in varied environments and how geographic distributions shape the functional traits in animals.

## CONFLICT OF INTEREST

The authors declare that there is no conflict of interest.

## AUTHOR CONTRIBUTIONS

**Yingjie Ma:** Conceptualization (equal); Data curation (lead); Formal analysis (lead); Methodology (lead); Writing‐original draft (lead); Writing‐review & editing (lead). **Meng Wang:** Formal analysis (equal); Methodology (equal); Software (equal); Visualization (equal); Writing‐review & editing (equal). **Fuwen Wei:** Conceptualization (equal); Project administration (equal); Supervision (equal); Writing‐review & editing (equal). **Yonggang Nie:** Conceptualization (lead); Project administration (lead); Supervision (lead); Writing‐review & editing (equal).

## Supporting information

Table S1

## Data Availability

No data are associated with this manuscript.
